# YOLO-ED: An efficient lung cancer detection model based on improved YOLOv8

**DOI:** 10.1371/journal.pone.0330732

**Published:** 2025-09-04

**Authors:** Qingqiang Zeng, Tao Hu, Zijie Chen, Jiexin Zheng, Jianqing Li, Yuanyuan Pan

**Affiliations:** 1 School of Computer Science and Engineering, Macau University of Science and Technology, Macau, China; 2 Central Laboratory, the Third People’s Hospital of ZhuHai, ZhuHai, China; Shijiazhuang Tiedao University, CHINA

## Abstract

In recent years, You Only Look Once (YOLO) models have gradually been applied to medical image object detection tasks due to their good scalability and excellent generalization performance, bringing new perspectives and approaches to this field. However, existing models overlook the impact of numerous consecutive convolutions and the sampling blur caused by bilinear interpolation, resulting in excessive computational costs and insufficient precision in object detection. To address these problems, we propose a YOLOv8-based model using Efficient modulation and dynamic upsampling (YOLO-ED) to detect lung cancer in CT images. Specifically, we incorporate two innovative modules, the Efficient Modulation module and the DySample module, into the YOLOv8 model. The Efficient Modulation module employs a weighted fusion strategy to extract features from input CT images, effectively reducing model parameters and computational overhead. Furthermore, the DySample module is designed to replace the conventional upsampling component in YOLO, thereby mitigating information loss when expanding feature maps. The dynamic bilinear interpolation introduced by this module increases random bias, which helps minimize errors in feature extraction. To validate the effectiveness of YOLO-ED, we compared it with baselines on the LUNG-PET-CT-DX lung cancer diagnosis dataset and the LUNA16 lung nodule dataset. The results show that YOLO-ED significantly improves precision and reduces computational cost on these two datasets, demonstrating its superiority in the detection of medical images.

## Introduction

Lung cancer is a prevalent disease known for its high mortality rate [1]. Computed tomography (CT) scanning is the most widely used technique to identify lung cancers [2,3]. People who are at high risk for lung cancer need to undergo regular CT screening to detect cancer in its early stages. Radiologists identify suspicious lesions in the form of pulmonary nodules on chest CT images, and early detection of patient symptoms improves the cure rate. Experienced physicians diagnose lung cancer according to the morphological features of the lesions on CT scans. Doctors confirm the presence of lung tumors by examining CT images for the presence of masses, nodules, cavities, or irregular shadows. The morphology and borders of the nodule are important factors in the imaging diagnosis of lung cancer. Malignant lesions usually have irregular or lobulated margins, while benign nodules may have clear and rounded borders. At the same time, the physician will focus on the nodule density and cavitation of the image. A low density area in the center of the nodule indicates that the area may be necrotic. Cavity formation in the lungs may be associated with certain types of cancer. Due to the complex vascular structure of the lungs, potential malignant pulmonary nodule lesions are easily overlooked. The skills and experience level of radiologists affect the effectiveness of lung lesion detection.

To assist physicians in cancer diagnosis, deep learning models are applied to identify categories of lung cancer [4]. Current deep learning-based lung cancer detection methods include Region-Convolutional Neural Networks(R-CNN) [5,6], Convolutional Neural Networks (CNN) [7–9], YOLO [10–12] and other models.The R-CNN based methods necessitate independent processing of numerous candidate regions in CT images, resulting in high computational costs and slow detection speeds, making it difficult to meet clinical diagnostic requirements. The CNN-based methods require a large number of network layers to improve the precision, which demands significant computational resources, making training and deployment difficult in resource-constrained environments. Compared with the previous two types of methods, the YOLO series model combines the features extracted by the earlier layers with those output by the subsequent layers, rather than simply concatenating the convolutional layers together. This holistic structure requires fewer computational resources while achieving higher precision. For instance, YOLOv5 extracts features by successive convolutional layers, fuses them, and utilizes anchored boxes to predict the type of lung cancer. Despite reducing computational overhead, YOLOv5 has been observed to be susceptible to inaccuracies in detecting tiny lung nodule targets. Conversely, the YOLOv8 model equipped with Anchor-Free performed well in detecting small lung targets. The length and width of the anchor frame adjusted with the size of the detected target automatically. By analyzing the model detection process, we discovered that YOLOv8 demands considerable computational resources. To reduce the computational cost of deep learning models, the most direct method is to use models with fewer parameters. However, this way typically results in a decreased in model precision, which is unacceptable in medical diagnosis due to its stringent precision demands. Therefore, our challenge lies in reducing the computational cost of the model while striving to enhance its precision.

Analysis of the YOLOv8 architecture indicates that multiple convolutional layers within the Cross Stage Partial Connections Bottleneck with 2 Convolutions (C2F) [12] module contribute significantly to the increased computational burden of the model. Additionally, the bilinear interpolation used in the upsampling layers of YOLOv8’s neck introduces limitations to the model’s precision. This approach often results in artifacts and smooth edges, which degrade sharpness and detail in high-frequency regions. To address these challenges, we replace the C2F module with the Efficient Modulation module, which integrates the strengths of modulation mechanisms and depth-wise convolution. Focal modulation mechanisms exhibit dynamic adaptability and an enlarged receptive field, enabling more effective feature interaction across varying spatial scales. Depth-wise convolution (DWConv) reduces both the number of parameters and the computational cost. Meanwhile, we substitute the upsampling layers [12] in YOLOv8 with DySample to prevent redundant offsets in dynamically selected regions. DySample effectively avoids the adverse effects of the checkerboard lattice effect [13]. These methods contribute to improved performance and precision in lung cancer object detection tasks while maintaining a lower computational cost.

In summary, the main contributions of this paper are as follows:

1. We present the YOLO-ED model, tailored for lung cancer detection, which incorporates an optimized architecture to enhance feature extraction and detection precision. This model addresses the critical demands of medical imaging by ensuring high precision and offering a lightweight architecture, making it a valuable tool for clinical applications.
2. We introduce the efficient modulation module as a replacement for the C2F module in the backbone of YOLOv8. This approach leverages attention mechanisms to reduce the number of convolutional layers, effectively decreasing the computational cost of the model while maintaining its precision. The precision of the model was enhanced by substituting the upsampling process within the YOLOv8’s neck section with the DySample technique.
3. To validate the effectiveness of our proposed method, we conducted comparative and ablation experiments. The experimental results demonstrate that YOLO-ED achieves the precision of 90.7% and reduces the computational load of 2.4GFLOPs. This superior performance highlights the robustness and efficacy of our approach in addressing the challenges of lung cancer detection.

The remainder of the paper is organized as follows. The Related Work section reviews the existing literature. The Methodology section details our proposed approach. The Experiment section describes the experimental setup, and the Results section presents the experimental results. Finally, the Conclusion section summarizes the main findings and contributions.

## Related work

### Lung cancer diagnosis

CT lung imaging features encompass a range of critical characteristics that are essential for accurate medical diagnosis and analysis. These features include variations in shape, size, texture, and intensity, as well as spatial relationships and distribution patterns within anatomical structures [14]. Deep learning models are particularly adept at detecting these subtle radiological characteristics, enabling them to distinguish and accurately classify different types of lung cancer [15,16].

Lung cancer is categorized into two primary types: small cell lung cancer (SCLC) and non-small cell lung cancer (NSCLC), which is further divided into three subtypes (adenocarcinoma (ADC), squamous cell carcinoma (SCC), and large cell carcinoma (LCC)). Small cell lung cancer (SCLC) [17] presents as central lesions, frequently positioned around the bronchial structures within the central pulmonary regions. Despite their initial small size, these tumors possess a remarkable capacity for rapid expansion, attributable to their aggressive nature. This high invasiveness often results in early mediastinal lymph node enlargement and extensive metastatic spread, distinguishing SCLC from other types of lung cancer. The morphology of these lesions is typically irregular, with poorly defined margins, reflecting their tendency to infiltrate along bronchial pathways and vascular structures. Furthermore, SCLC demonstrates a rapid rate of progression, with significant morphological changes observable over short intervals. Large cell carcinoma (LCC) [18] typically manifests as large, solitary masses with irregular borders and can occur in any part of the lung, though they frequently favor peripheral locations. These tumors are known for their rapid growth and potential to invade adjacent structures. A notable feature of LCC is the occasional formation of cavitations due to internal necrosis, resulting in heterogeneous density patterns within the tumor mass. In contrast, adenocarcinoma (ADC) [19] is predominantly present as peripheral lesions, often appearing as small nodules or masses with spiculated margins and sometimes accompanied by ground-glass opacities (GGO). The presence of GGO is particularly indicative of early-stage or minimally invasive adenocarcinoma. Although ADC generally grows more slowly than other lung cancer types, it remains capable of significant invasion. In advanced stages, this type of cancer may demonstrate multiple nodular or patchy lesions, highlighting its potential for multifocality. Squamous cell carcinoma (SCC) [20] presents as central lesions located around the bronchial regions, and often manifests as large, irregular masses with indistinct margins. A hallmark feature of SCC is the formation of cavitations within the tumor, particularly evident in larger lesions. In addition, these tumors can exhibit irregular calcifications. The aforementioned characteristics underscore the aggressive clinical behavior of lung cancer and highlight the importance of timely diagnosis and intervention.

### Deep learning for object detection methods

Deep learning models have been increasingly applied to the identification and classification of lung cancer categories. Han et al. [21] classified the PET/CT data of NSCLC patients using the Visual Geometry Group16 (VGG16) model. NSCLC can be classified according to histological subtype into ADC and SCC. Research indicates that the VGG16 model achieved an average precision of 84% in the classification task for NSCLC. DenseNet [22] model achieved an precision of 90% in the classification task for ADC, LCC and SCC. The dataset consists of 3,940 CT images from patients at Shandong Provincial Hospital in China. Jacob et al. [23] proposed a CNN-based no-reference image spatial quality evaluation algorithm. The algorithm achieved a precision of 93% in the lung cancer PET/CT image classification task. Barbouchi et al. [24] proposed the Detection Transformer (DERT) model to assist physicians in classifying stages of lung cancer. The experimental results indicate that the model achieved a precision of 93%. Wei et al. [25] introduced YOLOv5, which achieved the precision rate of 90% to identify lung cancer from diagnostic images. The Spatial Pyramid Pooling Fast (SPPF) module plays a pivotal role in enhancing detection precision. Currently, the precision of these models remains suboptimal for medical diagnostic applications. The inherent complexity and variability of medical data require a higher degree of precision to ensure reliable and safe clinical outcomes. Consequently, further enhancements in model performance are imperative. Furthermore, through a comparison with these studies, we observed that previous studies often neglected to perform experiments with patient stratification. This oversight poses a potential risk of patient leakage from the training set into the test set, which could lead to inflated performance results. To address the limitations identified in previous literature, the experiments in this study will involve processing the dataset on a per-patient basis.

### Efficient methods for object detection

Incorporating attention mechanisms into YOLOv8 offers significant benefits by enhancing its object detection performance. Firstly, attention mechanisms enable the model to focus on relevant features while suppressing irrelevant background noise, thereby improving detection precision. Secondly, these mechanisms facilitate adaptive feature weighting, allowing the model to dynamically prioritize spatial and channel information critical for identifying various objects. Attention mechanisms have found widespread application in object detection, such as Convolutional Block Attention Module (CBAM) [26], Squeeze-and-Excitation (SE) [27], and Efficient Channel Attention (ECA) [28]. However, one prominent drawback is the increased computational overhead they introduce, as attention layers add complexity and require additional resources for both computation and memory, potentially impacting model efficiency and speed. To address the excessive computational cost associated with traditional attention mechanisms, Yang introduced the Focal Modulation Network (FocalNet) [29]. This model replaces conventional self-attention mechanisms with an innovative Focal Modulation module, which enhances token interaction capabilities while maintaining computational efficiency. In addition, Guo reported that Depth-wise Convolution (DWConv), which is employed in the Visual Attention Network (VAN) [30] significantly reduces both the parameter count and computational complexity of the model. Thereby, we introduce the Efficient Modulation method (EM) [31], which integrates the advantages of VAN and FocalNet. EM effectively combines DWConv with modulation techniques, thereby significantly reducing the computational overhead typically incurred by attention layers. Furthermore, DySample [32] demonstrates significant potential for improving model precision by dynamically adjusting the sampling process based on the importance of input features.

## Methodology

### YOLOv8 algorithm

**Fig 1:**
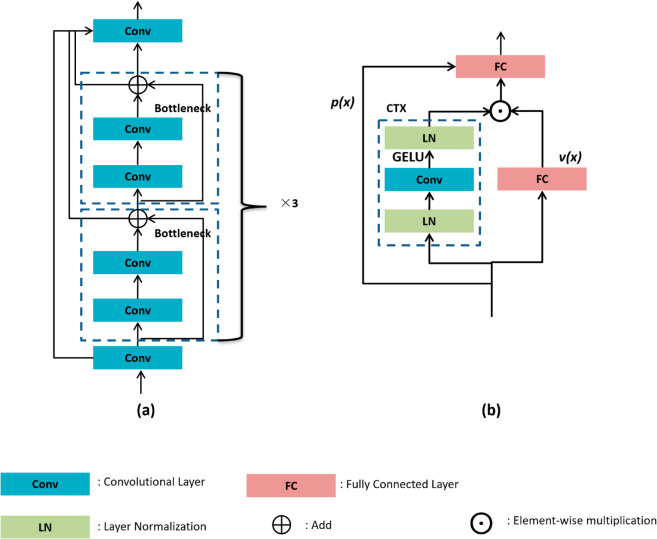
(a) C2F module (b) Efficient modulation module.

As mentioned above, many YOLO series models, such as YOLOv3, YOLOv5, YOLOv7 and YOLOv8, are used for lung cancer detection. Compared to YOLOv3, YOLOv8 adopts an innovative anchor-free design that simplifies multi-scale lung nodule detection in CT scans while delivering faster inference speeds. In contrast to YOLOv5, YOLOv8 introduces a residual architecture, enhancing the model’s gradient flow through additional skip connections and split operations. This modification significantly improves both the convergence speed and the overall performance of the model. When compared to YOLOv7, YOLOv8 incorporates a distinctive cross-stage partial (CSP) concept, thereby augmenting the model’s capacity for feature extraction and representation. Therefore, YOLOv8 is more suitable than other YOLO models for lung cancer detection based on CT images. The architecture of YOLOv8 comprises three components: backbone, neck and head. The backbone incorporates the CSP concept. The CSP structure consists of multiple C2F modules and convolutional cross-stage connections. C2F module employs a channel-halving strategy in each bottleneck module to reduce feature dimension. Fig 1(a) shows that the C2F module contains six bottleneck modules. Each bottleneck module contains two convolutional layers. The bottleneck modules are connected by residual. The output feature maps from multiple bottleneck modules are then concated with the original feature maps. The neck of YOLOv8 consists of SPPF and Path Aggregation Network (PANet) [33]. SPPF consists of two convolutional layers and three max-pool layers. The feature map is converted to a fixed-size feature vector by upsampling bilinear interpolation. Feature vectors are subjected to max-pooling operations at different scales. Then, the resulting image features are concatenated. The neck component utilizes PANet, this bidirectional pathway network enhances feature propagation capabilities by adding bottom-up sampling. In addition, PANet facilitates upward movement at a lower resolution and enrich feature details. The head part of YOLOv8 is mainly responsible for generating detection and target classification.

### Efficient modulation

As mentioned previously, the C2F module has many consecutive convolutional layers, and this increases the computational effort. To address this problem, we apply an efficient modulation module to replace the C2F module in backbone for feature extraction. The fully connection layers (FC) and modulation block are fused as shown in Fig 1(b). We leverage *p*(*x*) to compress the number of *v*(*x*) channels. We adapt the contextual modeling branch to improve efficiency. The process of the modulation mechanism is as follows: a large kernel convolutional block contextualizes the image vectors and then multiplies and modulates each element of the input features. A linear function *f*(*x*) maps the features of the input *x* to a new feature space. Local spatial information is modeled by deep convolution and GELU activation. We set the kernel size to 7 and the dilation to 1. This achieves a balance between model’s efficiency and receptive field size [31]. Finally, we employ a linear projection *g*(*x*) for channel communication. The context modeling branch *ctx*(*x*) is represented as follows:

$$ctx(x)=g(act(DWConv_{7.1}(f(x))))$$ (1)

The other branch performs the constant mapping. We use element-by-element multiplication to fuse the features of the two branches and subsequently add an FC layer *p*(*x*).

Output=p(ctx(x)⊙v(x)) (2)

Although the C2F module plays a critical role in multi-scale feature fusion, the replacement Efficient Modulation module has superior effectiveness which introduces a parallel dual-branch design. One branch focuses on context modeling, adaptively aggregating contextual information from different levels, while the other branch performs a linear projection to map features into a new space. These two parallel branches extract features from different feature spaces independently. The function of this module is similar to the self-attention mechanism, enabling the model to capture intricate details with greater precision.

### DySample

**Fig 2:**
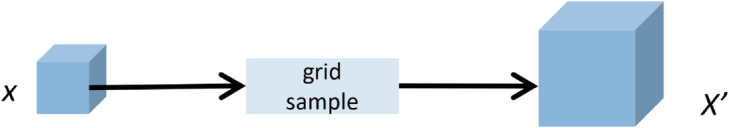
Structure of Bilinear interpolation upsample, input feature map (X), upsampled feature map (X’).

The YOLOv8’s upsample is a bilinear interpolation. The interpolated points are numerically estimated with four-pixel values on the *x*, *y* axes. The weights of the interpolated points are assigned according to their distance from these four points. The obtained weight values fill in the unknown pixel points. *X* represents input feature map, *S* represents sampling set and $X^{\prime }$ represents upsampled feature map. As shown in Fig 2, this process can be given by:

$$X^{\prime }=grid\_sample(X,S)$$ (3)

**Fig 3:**
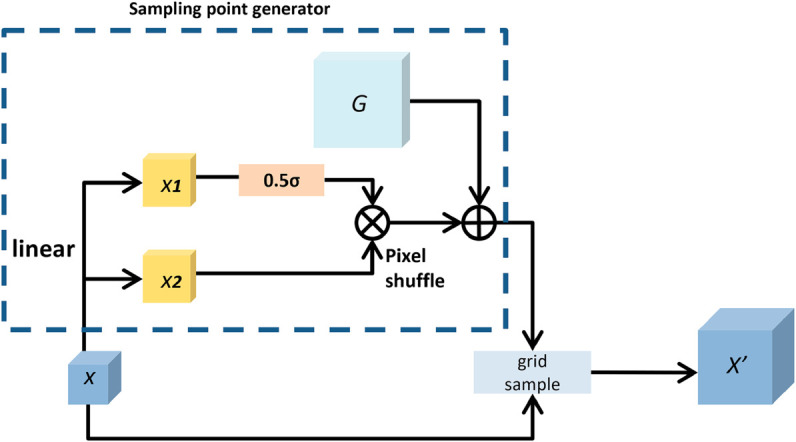
Structure of DySample original grid (G).

To enhance the model’s ability to manage spatial details and improve overall feature map resolution, we propose substitution of YOLOv8’s upsample layers with the DySample module, which integrates random bias and pixel shuffling mechanisms in this section. In Fig 3, we generate point-wise dynamic range factors by linearly projecting the input features. This approach increases the flexibility of the offset. By utilizing the sigmoid function along with a static factor of 0.5, the dynamic range is limited to values within [0, 0.5]. *O* represents the offset, which is generated as follows:

$$O=0.5\times sigmoid(linear_{1}(X))\cdot linear_{2}(X)$$ (4)

The offset set is generated by a linear projection. The offset set is reshaped to meet the spatial size requirements. This process involves pixel shuffling. Each sampling point in the sampling grid *G* is increased by a fixed offset, resulting in a new sampling set *S*. Finally, the upsampled feature map is generated with the sampling set by grid sample as Equation:

S=G+O (5)

### YOLO-ED

**Fig 4:**
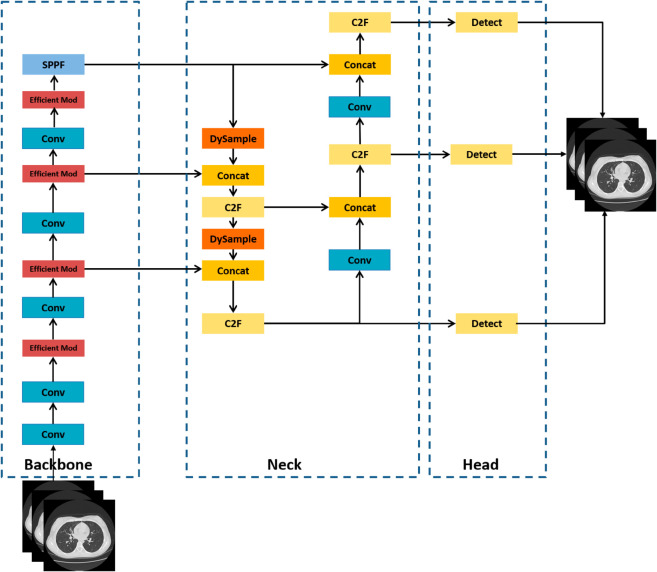
The structure of YOLO-ED.

In CT images, lung nodules have different imaging characteristics. ADC and SCC lung nodules are similar in diameter and size. The edges of the ADC and SCC lung nodules differ in terms of burr and roughness. They are categorized into two distinct types based on their radiological characteristics: grinding glass-like shadows and solid density. We propose the YOLO-ED model, an improvement over YOLOv8, designed to assist physicians in lung cancer detection. This new model not only improves detection efficiency but also maintains good detection precision. Specifically, the YOLO-ED model mainly makes two improvements to the original YOLOv8. First, the C2F modules in backbone are replaced by the Efficient Modulation module, which reduces the computational effort by the attention mechanism and decreases the number of convolutional layers. Second, the upsample layer on the neck part is replaced by the DySample module. DySample is dynamic bilinear interpolation. Additionally, it limits the range of dynamically generated offsets, thus reducing artifacts caused by sample overlap. The precision of model detection is improved by replacing the DySample module. Fig 4 clearly illustrates all the structures and details of YOLO-ED.

## Experiment

### Dataset

We conducted experiments on two publicly available datasets: Lung-PET-CT-DX and LUNA16. Lung-PET-CT-DX [34] is a publicly available multimodal data set designed for comprehensive research on various aspects of lung cancer. In this experiment, total of 204 patients were categorized into four distinct groups based on their histopathologic diagnoses: 100 cases of adenocarcinoma (ADC), 61 cases of small cell lung cancer (SCLC), 38 cases of squamous cell carcinoma (SCC), and 5 cases of large cell carcinoma (LCC). Patients within each category were divided into training, validation, and test sets using a 6:2:2 ratio. For each patient, 30 CT images were randomly selected and assigned exclusively to a single subset, ensuring no overlap between training, validation, and test sets. Table 1 summarizes the distribution of patients and CT scans across the training, validation, and test sets in the LUNG-PET-CT-DX dataset for the four lung cancer subtypes. Specifically, the test set includes 20 patients with ADC, 7 with SCLC, 1 with LCC, and 12 with SCC. After three junior radiologists labeled each image, two other senior radiologists reviewed them. The labeled files were converted to the Visual Object Classes (VOC) format for use in deep learning models. For training purposes, the three-channel CT images are converted into a single-channel format.

**Table 1:** LUNG-PET-CT-DX diagnosis dataset partition.

	ADC		SCLC		LCC		SCC	
	Patient	CT scan	Patient	CT scan	Patient	CT scan	Patient	CT scan
Train	60	900	24	720	3	90	37	1110
Valid	20	600	7	210	1	30	12	360
Test	20	600	7	210	1	30	12	360

To comprehensively evaluate the detection and recognition capabilities of the YOLO-ED model on diverse lung imaging datasets, we re-trained the model using the LUNA16 dataset. The LUNA16 [35] is a curated subset of the publicly available LIDC-IDRI dataset [36], which comprises 1,018 low-dose lung CT scans. From this dataset, 888 scans were selected to form LUNA16. Radiologists categorized lesions with a nodule diameter of ≥ 3 mm as potentially malignant, associating them with lung cancer. Four experienced radiologists annotated the locations and diameters of all detected nodules. For training purposes, the dataset was randomly split into training, validation, and test sets using a fixed random seed (seed=0), following a 6:2:2 ratio. Table 2 presents the partitioning of the LUNA16 dataset into training, validation, and test sets. Specifically, 532 CT scans were allocated to the training set, 178 scans to the validation set, and the remaining 178 scans to the test set.

**Table 2:** LUNA16 diagnosis dataset partition.

	Train	Valid	Test
CT scan	532	178	178

### Data augmentation

We used mosaic and Mixup data augmentation. Mosaic data augmentation concatenated four images into one image, which greatly enriches the background of the detected object. Mixup data augmentation is an algorithm utilized in computer vision for hybrid enhancement of images, which mixes different images to expand the training dataset.

### Training environment and evaluation indicators

We implemented our method via PyTorch [37]. All experiments were performed on an NVIDIA GeForce GTX 4090 GPU. The dimensions of each image are 512 × 512 pixels. To minimize training time and improve model convergence, we initialized the YOLO-ED model using officially pre-trained weight: https://github.com/111sadf/YOLO-ed/blob/main/yolov8-ed.pt. The training parameters are shown in Table 3.

**Table 3:** Training parameters.

Input Image Size	Batch Size	Momentum	Initial Learning Rate	Decay Index	Epochs
512 ×512	32	0.9	0.01	0.0005	300

Detection precision and computational volume are important measures of model performance. The precision (P), recall (R), average precision (AP) and mean average precision (mAP) are important indicators of detection precision [38]. Giga Floating Point Operations Per Second (GFLOPs) is a value that measures the amount of model computation. The calculation formulas are shown in Eqs 6–9:

$$P=\frac{TP}{TP+FP}$$ (6)

$$R=\frac{TP}{TP+FN}$$ (7)

$$AP=\int_{0}^{1}P(R)\;dR$$ (8)

$$mAP=\frac{1}{n}\sum^{n}AP_{i}$$ (9)

where *TP* represents the number of accurately predicted positive samples, *FP* represents the number of incorrectly predicted positive samples, and *FN* represents the number of incorrectly predicted negative samples, *n* represents the number of target categories to be detected, and *AP*_*i*_ represents the average precision for the *i*-th target category.

**Fig 5:**
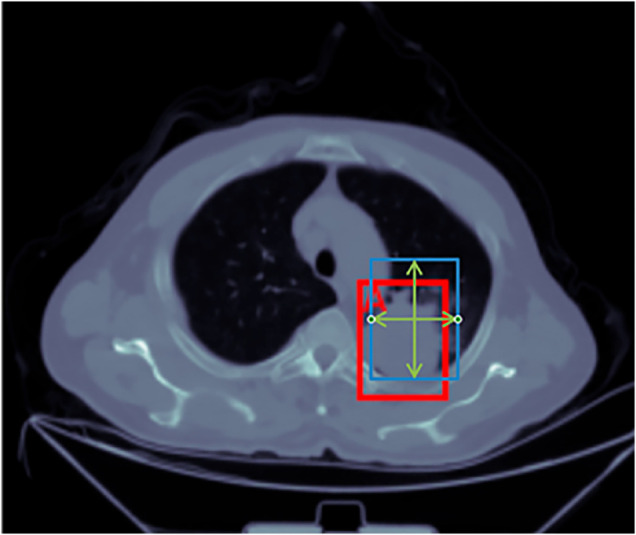
Prediction box and ground truth box.

The model’s output typically includes both bounding box positions and class probabilities. Bounding box positions refer to the predicted location and size of the detected object. In deep learning-based object detection, these positions are commonly represented using the center coordinates of the bounding box along with its width and height. As illustrated in Fig 5, taking the detection of nodules in ADC as an example, the red box represents the ground truth annotation, indicating the actual location of the nodule. In contrast, the blue box denotes the predicted bounding box generated by the model. Class probabilities refer to the likelihood that the detected object belongs to a specific category, as predicted by the model. To evaluate the precision of object localization, the Intersection over Union (IoU) metric is commonly used to measure the overlap between the predicted bounding box and the ground truth box. A detection is considered a true positive (TP) if the IoU is greater than or equal to 0.5. The calculation formulas are shown in Eq 10:

$$\text{IoU}=\frac{\left|A\cap B\right|}{\left|A\cup B\right|}$$ (10)

where A and B represent the predicted and ground truth regions, respectively. The numerator, |A∩B|, represents the area of overlap, while the denominator, |A∪B|, represents the combined area of both regions.

## Results

### Results of the enhanced backbone

To validate the efficacy of models with different attention mechanisms embedded, we have separately added four attention mechanisms to the backbone structure for Efficient Modulation (EM), SE, ECA, and CBAM to the backbone structure, while keeping other components unchanged. The modified models YOLO-EM, YOLO-SE, YOLO-ECA and YOLO-CBAM are tested and compared on the Lung-PET-CT-DX dataset, with the results presented in Table 4. From an attentional perspective, it can be seen that YOLO-EM reduces the amount of computational load by at least 12.2% compared to other attentional models, but the precision of the recognition (mAP@0.5:0.95) decreases by at most 0.01. These results indicate that incorporating the attention mechanism into the original YOLO model reduces computational load while maintaining detection accuracy. In particular, the EM mechanism demonstrates the most significant efficiency improvements among the tested attention mechanisms.

**Table 4:** Results of the integrating different attention modules on Lung-PET-CT-DX.

Model	P	R	mAP(@0.5)	mAP(@0.5:0.95)	GFLOPs
YOLOv8	0.879	0.871	0.873	0.525	8.9
YOLO-EM	0.890	0.878	0.889	0.537	**7.2**
YOLO-SE	0.887	0.876	0.881	0.535	8.3
YOLO-CBAM	0.888	**0.882**	0.886	0.543	8.3
YOLO-ECA	**0.891**	0.879	**0.890**	**0.547**	8.2

### Results of the enhanced neck

To verify the effectiveness of the upsample improvement, we replaced the original upsample layer in YOLOv8 with DySample while keeping all other structures identical. The detection results of the YOLO-DS model with DySample and the YOLOv8 model on the LUNG-PET-CT-DX dataset are shown in Table 5. It can be observed that, compared to YOLOv8, YOLO-DS achieves improvements in precision and recall by 1.90% and 1.80%, respectively. These results substantiate the effectiveness of the improved algorithm in enhancing model precision.

**Table 5:** Results of the improved upsample part on LUNG-PET-CT-DX.

Model	P	R	mAP(@0.5)	mAP(@0.5:0.95)	GFLOPs
YOLOv8	0.879	0.871	0.873	0.525	8.9
YOLO-DS	**0.898**	**0.889**	**0.893**	**0.539**	**8.2**

### Results of the enhanced backbone and neck

As previously mentioned, enhancing the backbone of YOLOv8 with an attention mechanism can reduce the model’s computational load, while enhancing the neck of YOLOv8 with DySample can improve the model’s recognition accuracy. To identify the optimal combination of the attention mechanism and DySample, we replace C2F with different attention mechanisms (EM, SE, ECA, and CBAM) and modify the upsample to DySample, resulting in four improved models: YOLO-ED, YOLO-SED, YOLO-ECD, and YOLO-CBD. Table 6 illustrates the performance of these improved models. It can be observed that the performance of all these models has improved compared to the original YOLOv8. Among them, YOLO-CBD attains the best mAP@0.5:0.95 of 54.80%, and YOLO-ED achieves the lowest computational cost of 6.5 GFLOPs. Obviously, in terms of both detection precision, recall rate and computational cost of model, YOLO-ED demonstrates a relatively balanced performance. Its precision is comparable to that of YOLO-CBD and YOLO-ECD; nevertheless, its computational cost is significantly lower. Therefore, YOLO-ED is selected to detect CT images of lung cancer.

**Table 6:** Results of the improved attention and upsample part on LUNG-PET-CT-DX.

Model	P	R	mAP(@0.5)	mAP(@0.5:0.95)	GFLOPs
YOLOv8	0.879	0.871	0.873	0.525	8.9
YOLO-SED	0.899	0.887	0.882	0.538	7.6
YOLO-CBD	0.903	0.899	0.891	**0.548**	7.6
YOLO-ECD	0.905	0.904	0.902	0.539	7.5
YOLO-ED	**0.907**	**0.904**	**0.905**	0.546	**6.5**

### Results of the ablation experiment

We perform a series of ablation experiments on the proposed YOLO-ED model, using YOLOv8 as a baseline. Table 7 presents the performance of the various components. When we replace the C2F module in the YOLOv8 backbone with the EM module, the computational load decreases by 1.7 GFLOPs, the number of training parameters is reduced by 0.25 M, and the precision increases by 1.1% compared to the baseline. These results indicate that replacing the C2F module with the EM module does not decrease precision, instead, it substantially reduces computational complexity. By replacing the traditional upsampling method with DySample, the precision of the model is improved by 1.9%, while the computational load is reduced by 0.7 GFLOPs. These results indicate that the DySample module contributes to improving precision. When EM is employed in the backbone network and DySample is incorporated into the neck network, the precision of the enhanced model reaches 90.7%. Concurrently, the model’s computational cost, measured in computational load, decreased to 6.5 GFLOPs, and the number of training parameters decreased to 2.53 M. Compared with the baseline model, this improvement results in a 2.8% increase in precision, a 2.4 GFLOPs reduction in computational load, and a 0.34 M decrease in the number of training parameters. In summary, the ablation experiments indicate that the introduction of the EM and DySample modules not only improves the model’s precision but also significantly reduces its computational cost.

**Table 7:** Ablation experimental results on LUNG-PET-CT-DX.

YOLOv8	EM	DySample	P	R	mAP(@0.5)	mAP(@0.5:0.95)	GFLOPs	Paramas
✓			0.879	0.871	0.873	0.525	8.9	2.87
✓	✓		0.890	0.878	0.889	0.537	7.2	2.62
✓		✓	0.898	0.889	0.893	0.539	8.2	2.78
✓	✓	✓	**0.907**	**0.904**	**0.905**	**0.546**	**6.5**	**2.53**

### Results of the comparative experiment

To further demonstrate the advantages of the algorithm introduced in this study. Faster R-CNN, Transformer, YOLOv3, YOLOv5, YOLOv7, YOLOv8 and YOLO-ED are validated on the LUNG-PET-CT-DX dataset and the LUNA16 dataset. Table 8 presents the results of the experiment on the LUNG-PET-CT-DX dataset. Compared with YOLOv8 model, the proposed YOLO-ED model achieves significant improvements in performance metrics, with increases of at least 2.80% in precision (P), 3.30% in recall (R), and 3.20% in mAP@0.5. Furthermore, it demonstrates improved efficiency, reducing computational complexity by at least 6.6 GFLOPs and the model size by 15.07 M parameters.

**Table 8:** Performance comparison of the different models on LUNG-PET-CT-DX.

Model	Faster R-CNN	DETR	YOLOv3	YOLOv5	YOLOv7	YOLOv8	YOLO-ED
P	0.798	0.831	0.823	0.850	0.845	0.879	**0.907**
R	0.802	0.862	0.827	0.822	0.858	0.871	**0.904**
mAP(@0.5)	0.819	0.846	0.843	0.851	0.865	0.873	**0.905**
GFLOPs	15.5	100.9	193.9	15.8	13.1	8.9	**6.5**
Params(M)	315.0	11.9	117.0	17.6	11.4	2.87	**2.53**

Similarly, as shown in Table 9, YOLO-ED achieves the best performance compared to existing models on the LUNA16 dataset, with parameter values of 92.6% for precision, 90.30% for recall, 94.7% for mAP@0.5, 6.5 GFLOPs for computational load and 2.53 M for the number of parameters.

**Table 9:** Performance comparison of the different models on LUNA16.

Model	Faster R-CNN	DETR	YOLOv3	YOLOv5	YOLOv7	YOLOv8	YOLO-ED
P	0.847	0.883	0.891	0.905	0.889	0.897	**0.926**
R	0.845	0.888	0.865	0.871	0.892	0.876	**0.903**
mAP(@0.5)	0.868	0.901	0.898	0.913	0.923	0.926	**0.947**
GFLOPs	15.5	100.9	193.9	15.8	13.1	8.9	**6.5**
Params(M)	315.0	11.9	117.0	17.6	11.4	2.87	**2.53**

**Fig 6:**
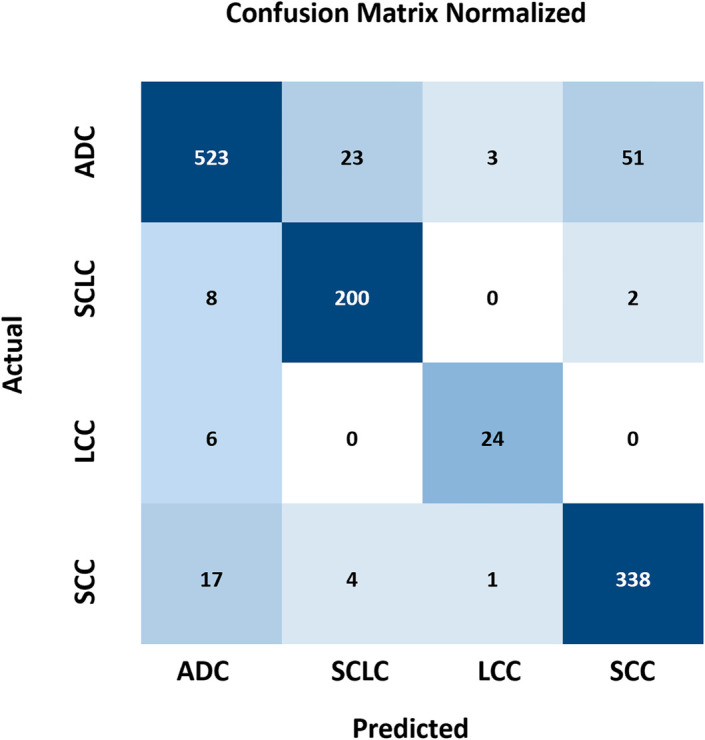
Lung cancer classification results on LUNG-PET-CT-DX.

**Fig 7:**
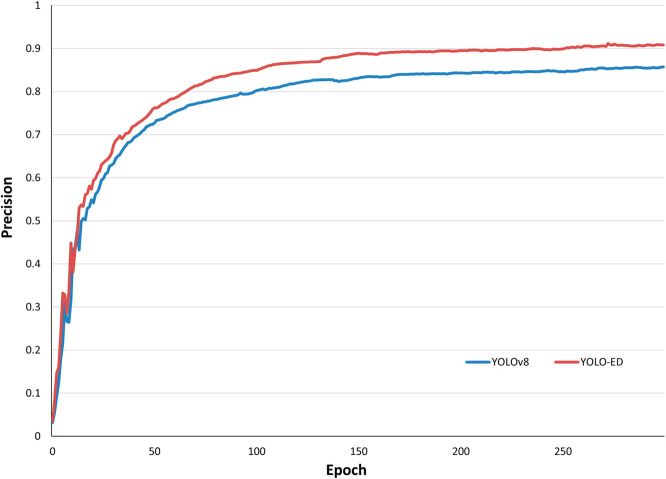
Precision change curve on LUNG-PET-CT-DX.

**Fig 8:**
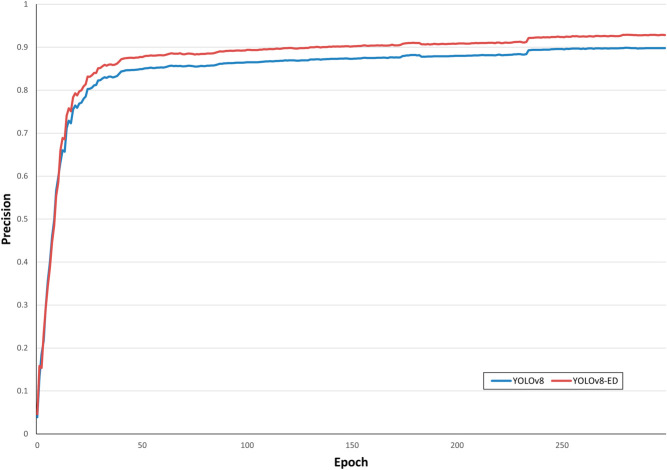
Precision change curve on LUNA16.

**Fig 9:**
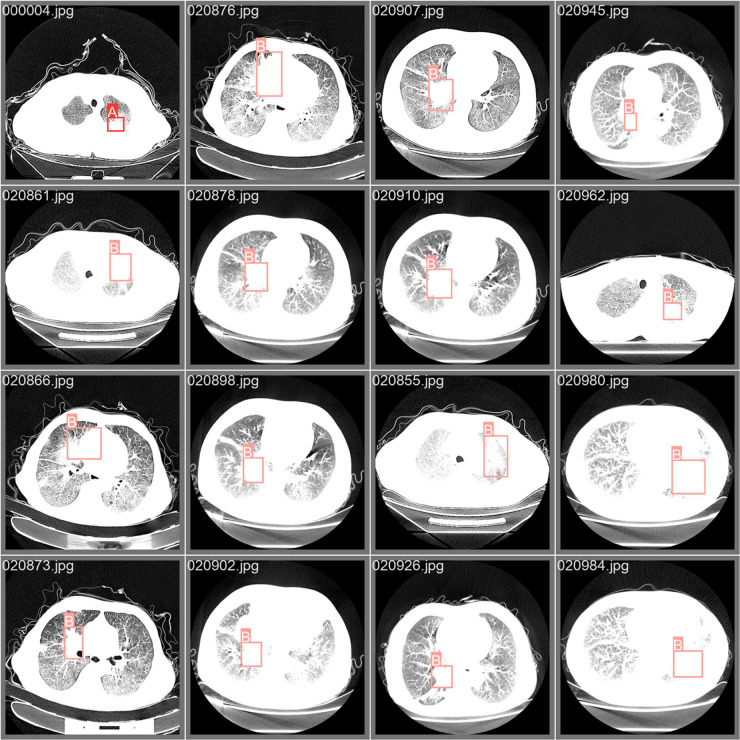
YOLO-ED detection effect on LUNG-PET-CT-DX.

**Fig 10:**
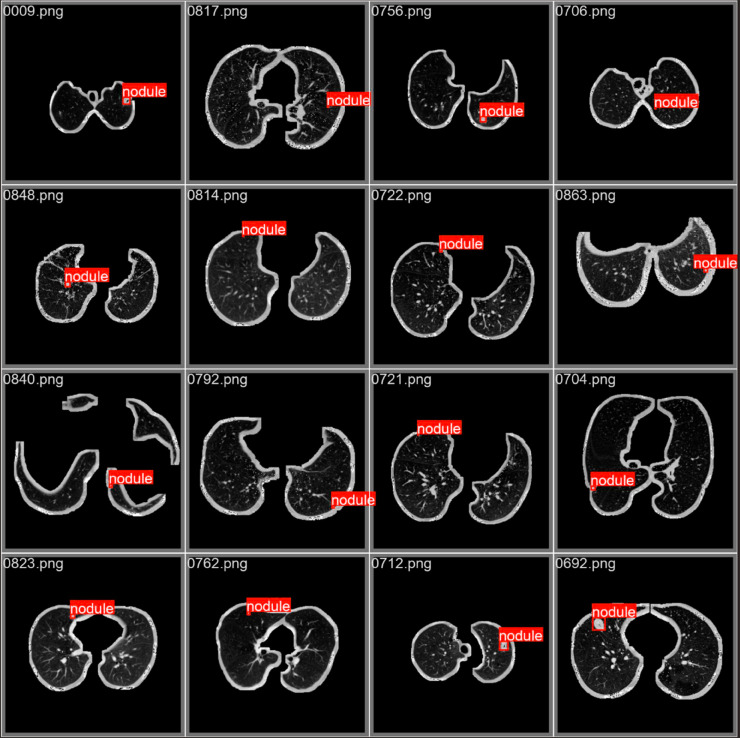
YOLO-ED detection effect on LUNA16.

To better illustrate the detection performance of YOLO-ED for different types of lung cancer, we evaluated the model using a normalized confusion matrix, as shown in Fig 6. Upon examining the experimental outcomes on the LUNG-PET-CT-DX dataset, YOLO-ED attained the precision of 94.4% for ADC, 88.1% for SCLC, 85.7% for LCC, and 86.4% for SCC. The model exhibited superior detection performance for ADC, SCLC and SCC, whereas its precision for LCC was comparatively lower. By analyzing the dataset, it was found that the reason for the low precision in LCC detection was the insufficient number of samples, namely, there were only 5 cases of LCC in the LUNG-PET-CT-DX dataset. Fig 7 shows the precision variation curve throughout the training process on the LUNG-PET-CT-DX dataset. The precision of YOLOv8 and YOLO-ED can reach 87.9% and 90.7%, respectively. In the early stages of training, the YOLO-ED precision rate is relatively low and highly variable. Fig 8 shows the convergence trends of YOLOv8 and YOLO-ED on the LUNA16 dataset, which are similar to those observed on the LUNG-PET-CT-DX dataset. Ultimately, the models achieved precision rates of 89.7% and 92.6%, respectively. Fig 9 presents the detection results of the YOLO-ED model on the LUNG-PET-CT-DX dataset, where ADC and SCLC are identified and labeled as A and B, respectively. The detected lung cancer lesions are clearly marked in the images. Fig 10 illustrates the detection results of the YOLO-ED model on the LUNA16 dataset, where pulmonary nodules are accurately identified and their locations are annotated in the images. In conclusion, previous quantitative comparisons indicate that our YOLO-ED is a practical and effective solution to the problem of lung cancer identification.

### Discussion

In this study, we established a novel YOLO-ED algorithm based on a YOLO framework to detect lung cancer. By comparing the results of YOLO-ED recognition with the annotations made by experienced radiologists, we found that YOLO-ED can effectively identify the differences in the edges, textures, or densities of different types of lung cancer. In addition, the model has been proven to detect lung nodules and assess their malignancy, predicting the likelihood of the patient developing cancer. In early lung cancer screening, the YOLO-ED model can help physicians identify the type and location of cancer. When combined with clinical symptoms and pathological examination results, it can ultimately provide a clear diagnosis for the patient.

There are still some limitations in this study. For example, the Depthwise Convolution (DwConv) used in the Efficient Modulation module may lead to a lack of spatial correlation in the model. Since DwConv performs feature extraction independently across different channels, it results in the extracted features being less comprehensive and potentially missing important inter-channel relationships. In the future, we will consider adding channel convolutions or spatial convolutions in the Efficient Modulation module. This would enhance the model’s ability to integrate features across multiple channels and enable the extraction of more comprehensive and richer information.

## Conclusion

This paper proposed a lung cancer detection model based on YOLOv8, called YOLO-ED. In this model, we integrated the Efficient Modulation module with YOLOv8, effectively reducing the model’s computational complexity while ensuring detection precision. On the other hand, we applied the DySample module to YOLOv8, which further enhances the model’s precision. The experimental results show that YOLO-ED achieves a precision of 90.7% on the LUNG-PET-CT-DX dataset and 92.6% on the LUNA16 dataset, respectively, all while reducing the GFLOPs by 2.4 compared to existing models. This indicates that our proposed model outperforms existing models, providing an effective solution for medical image detection.

The YOLO-ED model demonstrates significant potential for real-world deployment, particularly in resource-constrained environments. Its lightweight architecture enables efficient inference on low-cost devices, addressing the hardware limitations faced by community clinics. Additionally, YOLO-ED maintains precision comparable to manual review while ensuring efficiency. This capability assists primary care physicians in quickly identifying suspicious nodules, thereby reducing the workload of radiologists.
